# Synergistic changes in bystander CD8 and conventional CD4 T cells during neoadjuvant chemoimmunotherapy for non-small cell lung cancer reveal treatment response

**DOI:** 10.3389/pore.2025.1612229

**Published:** 2025-10-28

**Authors:** Li Wu, Liying Yang, Jian Sun, Miaoqing Zhao, Jiaxiao Geng, Fanghan Cao, Qianhui Chen, Yushan Yan, Hao Yang, Xiaorong Sun, Ligang Xing

**Affiliations:** ^1^ Department of Oncology, The Affiliated Hospital of Southwest Medical University, Southwest Medical University, Luzhou, Sichuan, China; ^2^ Shandong University Cancer Center, Shandong University, Jinan, Shandong, China; ^3^ Department of Radiation Oncology, Shandong Cancer Hospital and Institute, Shandong First Medical University, and Shandong Academy of Medical Sciences, Jinan, Shandong, China; ^4^ Department of Thoracic Surgery, Shandong Cancer Hospital and Institute, Shandong First Medical University and Shandong Academy of Medical Sciences, Jinan, Shandong, China; ^5^ Department of Pathology, Shandong Cancer Hospital and Institute, Shandong First Medical University and Shandong Academy of Medical Sciences, Jinan, Shandong, China; ^6^ Shandong Cancer Hospital and Institute, Shandong First Medical University and Shandong Academy of Medical Sciences, Jinan, Shandong, China; ^7^ Department of Nuclear Medicine, Shandong Cancer Hospital and Institute, Shandong First Medical University and Shandong Academy of Medical Sciences, Jinan, Shandong, China

**Keywords:** non-small cell lung cancer, neoadjuvant chemoimmunotherapy, bystander CD8, conventional CD4, hypoxia inducible factor-1α

## Abstract

**Objective:**

We analyzed changes in intratumoral CD8^+^ and CD4^+^ T-cell subpopulations following neoadjuvant chemoimmunotherapy in non-small cell lung cancer. We then assessed whether these alterations favored better outcomes and explored their association with the tumor microenvironment.

**Methods:**

Paired pre- and post-treatment samples from 32 patients with non-small cell lung cancer who underwent neoadjuvant chemoimmunotherapy at Shandong Cancer Hospital (January 2021–June 2023) were analyzed retrospectively. A quantitative analysis of tumor cells and their microenvironment was performed using a tissue microarray and a multiplex immunofluorescence technique. The analysis was based on the number of cells per thousand nucleated cells. Patients exhibiting a major pathologic response were classified as responders. The delta parameter (post-treatment minus pre-treatment) was utilized to assess changes in these indicators, and associations with treatment response were identified using the Wilcoxon Signed-Rank test and logistic regression analyses.

**Results:**

Of the 32 patients, 59.38% were classified as responders. Across all patients, neoadjuvant chemoimmunotherapy significantly reduced the densities of dysfunctional CD8^+^ resident memory T cells and cytotoxic and dysfunctional CD8^+^ bystander T cells, while conventional CD4^+^ T cells increased significantly. Similar trends were observed in the response group. In the non-response group, only cytotoxic CD8^+^ bystander T cells were reduced in number. Logistic regression analysis revealed that a high delta conventional CD4^+^ T cells is more favorable for MPR (OR = 0.13, p = 0.038), exhibiting a similar trend to changes in HIF-1α (p = 0.049).

**Conclusion:**

Alterations in specific CD8^+^ and CD4^+^ T-cell subpopulations during neoadjuvant chemoimmunotherapy may favor better outcomes and are potentially associated with tumor hypoxia. These findings provide a new perspective on developing strategies to improve treatment sensitivity in non-small cell lung cancer.

## Introduction

Lung cancer is a major cause of cancer-related mortality worldwide, with non-small cell lung cancer (NSCLC) accounting for the majority (80%–85%) of cases. At the time of diagnosis, the 5-year overall survival rate is 18% [1–4]. In recent decades, a plethora of clinical trials and studies have reported that immunotherapy has revolutionized the treatment of NSCLC, resulting in pathological remission rates ranging from 18% to 83% [5–8]. Nevertheless, patient response to immunotherapy remains inconsistent, with less than half of patients demonstrating a durable response. Efforts to enhance the efficacy of immunotherapy are hindered by a paucity of knowledge regarding the properties of the cells that initiate anti-tumor immune responses [9]. Consequently, further elucidation of the changes and functional status of the diverse cellular components in the tumor immune microenvironment (TIME) could yield novel insights into enhancing the efficacy of immunotherapy and further exploration of biomarkers to predict therapeutic response.

Currently, several studies have demonstrated that CD8^+^ resident memory T cells (T_rm_) exhibit favorable prognoses and are associated with enhanced immunotherapy efficacy across a range of cancer types [10]. The role of T_rm_ in promoting cytotoxic killing responses has been demonstrated [11]. A study of 111 patients with advanced NSCLC treated with a single-agent anti-PD-(L)1 monoclonal antibody demonstrated that patients with highly infiltrated tumors with T_rm_ had longer progression-free survival [12]. Consequently, T_rm_ cells may serve as potential biomarkers when selecting patients who may benefit from immunotherapy. Within TIME, tumor antigen-specific T cells, which represent the primary force of anti-tumor immunity, account for a negligible proportion. In contrast, a substantial number of CD8^+^ bystander T cells (T_bys_) exist, which do not recognize tumor antigens but specifically recognize various types of viruses that the body has been infected with in the past [13–16]. Some studies suggest that T_rm_ and T_bys_ cells can be distinguished based on CD103 expression [17, 18]. Recent studies have found that T_bys_ have the characteristics of memory T cells, and Lilin Ye et al. used oncolytic virus vectors to deliver antigenic epitopes recognized by T_bys_ cells to tumor cells, thereby activating T_bys_ cells to recognize and kill tumors, effectively controlling tumor progression, and providing a new solution for the treatment of tumors [13]. In addition, bystander T cells are not a homogeneous group of cells, but are composed of heterogeneous cells in different functional states [15]. Nevertheless, the role of T_bys_ in immunotherapy remains to be elucidated. As tumors progress, prolonged tumor burden and stimulation can result in the sustained expression of inhibitory molecules on T cells, such as PD-1, LAG-3, TIM-3, CTLA-4, and KLRG1 [19–21]. This ultimately leads to T cell dysfunction. However, recent studies have confirmed that dysfunctional T cells do not constitute a distinct, clearly defined subgroup. At the very least, T cells cannot simply be categorized as either dysfunctional or non-dysfunctional (or exhausted or non-exhausted) [17]. Although the upregulation of programmed cell death-1 (PD-1) on T cells is now considered a key indicator of T cell dysfunction [20–22], research by Kyoo-A. Lee et al. suggest that TIM-3 expression is a better marker of severely dysfunctional CD8^+^ T cells [23]. Some studies suggest that the functional state of CD8^+^ T cells can be determined based on their expression of PD-1 and TIM-3 [17, 24, 25]. Based on the above studies, we define T cells that express PD-1 but not TIM-3 as pre-dysfunctional (CD8^+^PD-1^+^TIM-3^-^). T cells expressing TIM-3 are defined as terminally dysfunctional T cells (CD8^+^PD-1^±^TIM-3^+^), representing a more severe state of dysfunction, regardless of PD-1 expression. T cells that express neither PD-1 nor TIM-3 are defined as non-dysfunctional cytotoxic T cells (CD8^+^PD-1^−^TIM-3^-^).

Cytotoxic T cells are pivotal effectors of anti-tumor immunity. While CD8^+^ T cells are widely regarded as the primary target for immunotherapeutic interventions due to their well-established role in anti-tumor immunity, recent studies have identified that CD4^+^ T cells exhibit a cytotoxic program. CD4^+^ T cells can be targeted to tumor cells in a variety of ways, by both direct cytolytic mechanisms and indirectly by regulating the tumor microenvironment [26, 27]. Tomasz Ahrends et al. utilized a mouse model of an anti-tumor vaccine to investigate the molecular mechanisms underlying the effects of CD4^+^ T cells on cytotoxic T lymphocytes. Their research revealed that CD4^+^ T cells can regulate the expression of co-inhibitory receptors, thereby impacting the activity of cytotoxic T lymphocytes [28]. Additionally, they observed that CD4^+^ T cells can upregulate the expression of chemokine receptors on cytotoxic T lymphocytes, which in turn facilitates their migration towards tumor cells and subsequent recognition, ultimately leading to their demise. This process of migration and recognition by cytotoxic T lymphocytes is a crucial component in the anti-tumor immune response. Furthermore, single-cell sequencing of patients with stage IIIA NSCLC has demonstrated that the synergistic proliferation of B cells and CD4^+^ T cells is associated with a positive treatment response to neoadjuvant immunotherapy [29].

In this study, multiplex immunofluorescence (mIF) was utilized to characterize and quantify T_rm_, T_bys_, CD4^+^ T cells, and their subpopulations in TIME of pre- and post-treatment specimens from NSCLC patients. This approach enabled the exploration of changes during neoadjuvant chemoimmunotherapy and whether these changes favor a better response.

## Methods

### Patients and specimens

In this retrospective study, formalin-fixed paraffin-embedded (FFPE) tissue sections from 32 patients with NSCLC were examined. The patients underwent radical surgery between January 2021 and June 2023 at Shandong Cancer Hospital. They had received two to four neoadjuvant chemoimmunotherapy (NCIT) cycles until surgeons assessed them as ready for surgery. A total of 32 patients had pre-treatment puncture specimens and paired surgical resection specimens. The post-treatment tumor tissue was obtained from a surgical resection sample taken from the same lesion as the pre-treatment biopsy. The inclusion criteria were as follows: (1) primary NSCLC; (2) stage IIA ∼ IIIB resectable NSCLC; (3) receiving NCIT + radical surgery; (4) aged ≥18 years; and (5) having a pretreatment puncture specimen and a paired surgical resection specimen [6]. Patients meeting any of the following criteria were excluded: (1) a combination of other malignancies; (2) previous autoimmune disease; (3) lack of detailed clinical information; and (4) incomplete specimens [30].(Supplementary Figure 1) The study was approved by the Ethical Review Committee of Shandong Cancer Hospital, and written informed consent from patients is not required.

### Pathological assessment

Pathological response was assessed according to the International Association for the Study of Lung Cancer multidisciplinary recommendations for pathological evaluation of lung cancer resection after NCIT. Major pathologic response (MPR) was defined as the reduction of viable tumor to the amount beneath an established clinically significant cut-off, based on prior evidence according to the individual histologic type of lung cancer and a specific therapy, on review of hematoxylin and eosin slides after complete evaluation of a resected lung cancer specimen (including all sampled regional lymph nodes) [31]. Tumors exhibiting ≤10% viable tumor cells were designated as having MPR. Following initial clinical reporting, the responses were reviewed in a blinded manner by two experienced pathologists from Shandong Cancer Hospital, and the average scores were used for final analysis. In the present study, MPR patients were classified as responders (“response” group), while the remaining patients were classified as non-responders (“non-response” group).

### Tissue microarrays

A total of 32 FFPE surgically resected specimens from NSCLC were collected, and a pathologist reviewed the specimens using hematoxylin and eosin-stained slides. This was done to determine the quality of the sample, select representative tumor regions for tissue microarray construction [32], and perform histomorphometry analysis. Subsequently, four 1-mm diameter cores were extracted from the representative tumor region of each surgically resected tumor FFPE block [32, 33]. Three tissue microarray blocks were then created using up to four tissue cores (1 mm diameter) from each NSCLC surgically resected specimen for further mIF staining.

### Multiplex immunofluorescence staining

**FIGURE 1:**
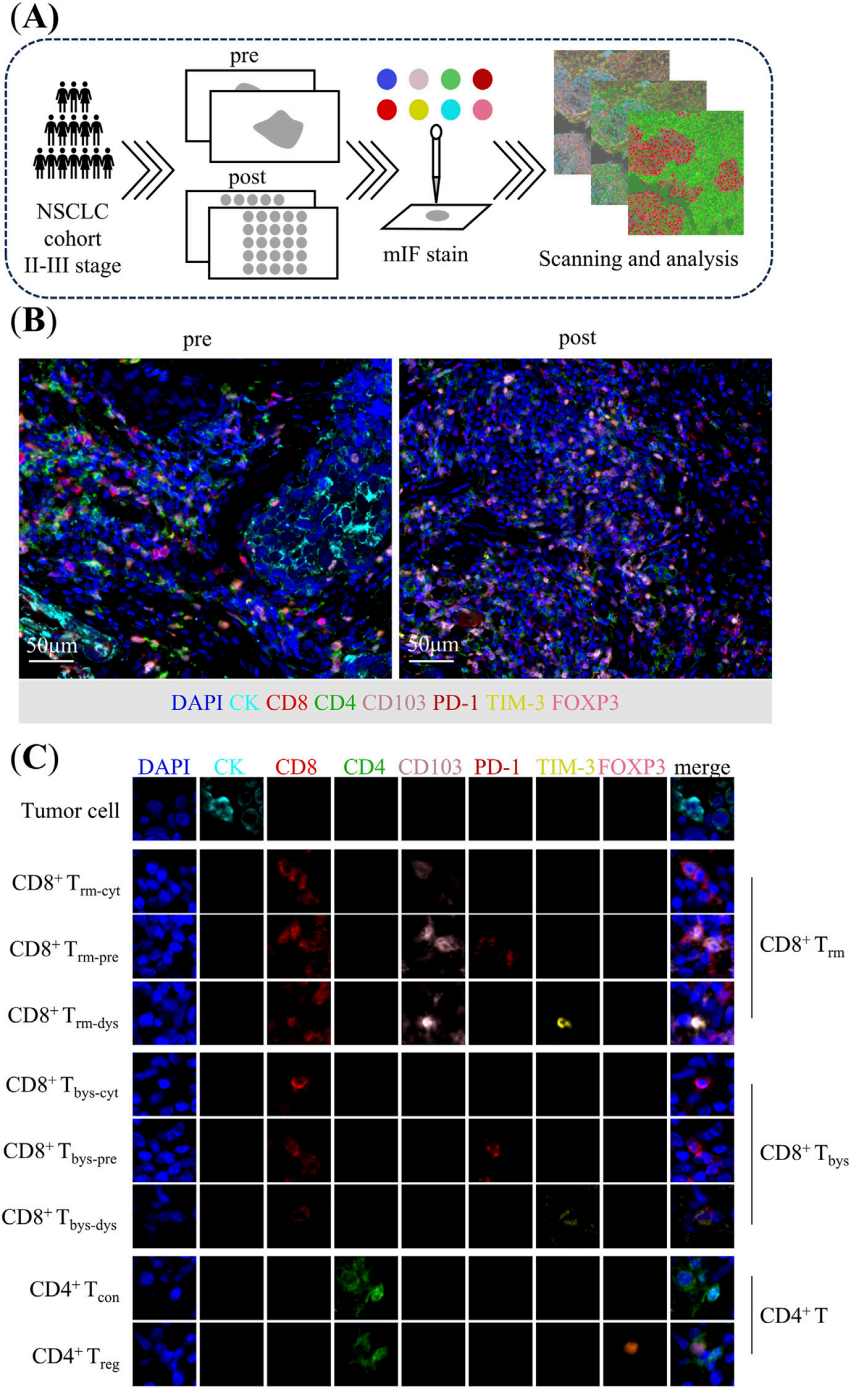
Analysis of tumor immune microenvironment in patients with non-small cell lung cancer. **(A)** Flow chart. **(B)** Multiplex immunofluorescence staining images of representative neoadjuvant chemoimmunotherapy pre-treatment puncture specimens and post-treatment surgical specimens. **(C)** Characterization of corresponding cells identified by co-expression of proteins in multiplex immunofluorescence images. CD8^+^ T_rm_: CK^−^CD8^+^CD4^−^CD103^+^PD-1^±^TIM-3^±^; CD8^+^ T_rm-cyt_: CK^−^CD8^+^CD4^−^CD103^+^PD-1^−^TIM-3^−^; CD8^+^ T_rm-pre_: CK^−^CD8^+^CD4^−^CD103^+^PD-1^+^TIM-3^-^; CD8^+^ T_rm-dys_: CK^−^CD8^+^CD4^−^CD103^+^PD-1^±^TIM-3^+^; CD8^+^ T_bys_: CK^−^CD8^+^CD4^−^CD103^−^PD-1^±^TIM-3^±^; CD8^+^ T_bys-cyt_: CK^−^CD8^+^CD4^−^CD103^−^PD-1^−^TIM-3^−^; CD8^+^ T_bys-pre_: CK^−^CD8^+^CD4^−^CD103^−^PD-1^+^TIM-3^-^; CD8^+^ T_bys-dys_: CK^−^CD8^+^CD4^−^CD103^−^PD-1^±^TIM-3^+^; CD4^+^ T: CK^−^CD8^−^CD4^+^; CD4^+^ T_con_: CK^−^CD8^−^CD4^+^FOXP3^-^; CD4^+^ T_reg_: CK^−^CD8^−^CD4^+^FOXP3^+^.

To perform a comprehensive analysis of the TIME of the puncture pretreatment specimens as well as the surgically resected post-treatment specimens, we performed mIF staining. NSCLC specimens (3-μm sections) were cut from the FFPE blocks and transferred onto positively charged slides, followed by mIF staining with the Opal 7-Color fIHC Kit (PerkinElmer, Waltham, MA) [34, 35]. The abbreviated workflow is as follows: Slides are baked overnight at 60 °C in an incubator, dewaxed using Leica Bond Dewax solution (#AR9222, Leica Biosystems), and then sequentially placed in 100%, 90%, and 75% ethanol. Antigen retrieval is performed for 20 min using Bond Antigen Retrieval Reagent 2 (#AR9640, Leica Biosystems). The slides are incubated with the primary antibodies (diluted according to the antibody instructions), followed by the addition of the horseradish peroxidase-labelled secondary antibody. This process is repeated, with each slide being subjected to seven sequential rounds of staining. Finally, nuclei were labeled with DAPI staining. Whole slide scans were acquired using the ×10 objective via the Vectra imaging system (Vectra Polaris 1.0.10) (Figure 1A).

The antibody panel composed by CK (clone AE1/AE3, dilution 1:200; Zsgb-bio), CD8 (clone EPR22483-288, dilution 1:400; Abcam), CD4 (clone EPR6855, dilution 1:200; Abcam), CD103 (clone EPR22590-27, dilution 1:500; Abcam), PD-1 (clone, UMAB199, Working fluid; Zsgb-bio), TIM-3 (clone D5D5R, dilution 1:200; Cell Signaling Technology), FOXP3 (clone 236A/E7, dilution 1:100; Abcam) for panel 1. CK (clone AE1/AE3, dilution 1:200; Zsgb-bio), CD31 (clone EPR17259, dilution 1:2000; Abcam), hypoxia inducible factor-1α (HIF-1α) (clone EP1215Y, dilution 1:100; Abcam), α-SMA (clone 1A4, dilution 1:200; Abcam) for panel 2 (Figures 1B,C).

### Multispectral analysis

Multiplex-stained slides were scanned using the Vectra Polaris Automated Quantitative Pathology Imaging System (Akoya Biosciences, Marlborough, MA, USA) at 20 nm wavelength intervals from 440 to 780 nm with a fixed exposure time and a magnification of ×10 [36]. The regions of interest (ROIs) were carefully selected by a pathologist based on H&E slides and CK expression. We increased the area by 10% to encompass the entire tissue core, giving an ROI area of 1.13 mm^2^. For puncture and surgically resected specimens, two to three ROIs with evidence of tumor-associated microenvironment were selected for precise scanning at ×20magnification (Supplementary Figure 2).

Representative images for training were selected in Phenochart (Akoya Biosciences, Marlborough, MA, USA), and an algorithm was created in the inForm 2.4 Image Analysis software (Akoya Biosciences, Marlborough, MA, USA) [34, 35]. Multispectral images were unmixed using the spectral library in inForm software, and based on DAPI staining, every single cell was segmented, and phenotyping was performed according to the expression compartment and intensity of each marker. Batch analysis was performed on selected ROIs of all tissues using the same algorithm designed on representative images by the inForm Software. The exported data were consolidated and analyzed in R software using the phenoptr (Akoya Biosciences, Marlborough, MA, USA) and phenoptr Report packages (Akoya Biosciences, Marlborough, MA, USA).

The quantities of various cell populations were expressed as the number of stained cells per 1000 nucleated cells [37]. When analyzing the data, only the number of cells positive for markers was evaluated. To analyze the changes in the microenvironment during NCIT, we introduced the delta parameter, which was defined as the tumor-infiltrating lymphocytes (TILs) observed in the post-treatment specimens minus those observed in the paired pre-treatment specimens [34].

### Definition of cellular characterization

The study delineated the following definitions for various T cell categories: tumor cell as CK^+^CD8^−^CD4^−^; CD8^+^ T cell as CK^−^CD8^+^CD4^−^; CD8^+^ resident memory T cell (CD8^+^ T_rm_) as CK^−^CD8^+^CD4^−^CD103^+^PD-1^±^TIM-3^±^; cytotoxic CD8^+^ resident memory T cell (CD8^+^ T_rm-cyt_) as CK^−^CD8^+^CD4^−^CD103^+^PD-1^−^TIM-3^-^; pre-dysfunctional CD8^+^ resident memory T cell (CD8^+^ T_rm-pre_) as CK^−^CD8^+^CD4^−^CD103^+^PD-1^+^TIM-3^-^; dysfunctional CD8^+^ resident memory T cell (CD8^+^ T_rm-dys_) as CK^−^CD8^+^CD4^−^CD103^+^PD-1^±^TIM-3^+^; CD8^+^ bystander T cell (CD8^+^ T_bys_) as CK^−^CD8^+^CD4^−^CD103^−^PD-1^±^TIM-3^±^; cytotoxic CD8^+^ bystander T cell (CD8^+^ T_bys-cyt_) as CK^−^CD8^+^CD4^−^CD103^−^PD-1^−^TIM-3^-^; pre-dysfunctional CD8^+^ bystander T cell (CD8^+^ T_bys-pre_) as CK^−^CD8^+^CD4^−^CD103^−^PD-1^+^TIM-3^-^; dysfunctional CD8^+^ bystander T cell (CD8^+^ T_bys-dys_) as CK^−^CD8^+^CD4^−^CD103^−^PD-1^±^TIM-3^+^ [17–23]. CD4^+^ T cells as CK^−^CD8^−^CD4^+^; conventional CD4^+^ T cell (CD4^+^ T_con_) as CK^−^CD8^−^CD4^+^FOXP3^-^; regulatory CD4^+^ T cell (CD4^+^ T_reg_) as CK^−^CD8^−^CD4^+^FOXP3^+^ [38]. Two stroma components were defined: microvessel density (MVD) as CD31^+^ [39] and cancer-associated fibroblasts (CAFs) α-SMA^+^ [40]. HIF-1α, a hypoxia marker expressed on tumor cells, CD4, or CD8.

Based on the above markers, different cell types can be identified, and cell counts are expressed as the number of cells per 1000 nucleated cells. For quantitative analysis of CAF/MVD/HIF-1α, the number of cells expressing CAF^+^, MVD^+^, and HIF-1α^+^ was counted per 1000 nucleated cells. This study did not distinguish between tumor and stroma in cell counting.

### Statistical analysis

Categorical variables are expressed as frequencies and percentages, and continuous variables are expressed as medians and interquartile ranges. The Fisher’s exact test was used to compare differences in the distribution of clinical characteristics between response and non-response groups. The Mann-Whitney U test was employed to assess differences in cell density across different groups. The Wilcoxon Signed-Rank Test was used to compare TILs pre- and post-NCIT. In this study, the delta parameter was introduced to represent the change in TILs during NCIT (post-treatment minus pre-treatment), and a logistic regression test was used to compare the effect of the change in TILs on the efficacy of NCIT. All analyses were performed using SPSS (20.0) and R software (4.1.2). Additionally, the dot-style visualization was generated using R software’s “Spatial map viewer” to process TIFF images obtained through segmentation and characterization identification performed by the inForm software. A p-value less than 0.05 was considered statistically significant.

## Result

### The clinicopathologic details stratified by treatment response status

A total of 32 patients with NSCLC receiving NCIT were enrolled in this study, and all enrolled patients had paired pre- and post-treatment specimens. Of the 32 patients, 84.38% (27/32) of the patients were male, 53.12% (17/32) were over 65 years of age, and 62.50% (20/32) had a smoking index greater than 400 cigarettes per year. Furthermore, 59.38% (19/32) of the patients had a KPS of 90. The histology types were predominantly squamous cell carcinoma (26/32, 81.25%), and the pathologic stage was predominantly stage IIIA (13/32). Immunotherapy regimens that were utilized included sintilimab (7/32, 21.88%), toripalimab (6/32, 18.75%), camrelizumab (6/32, 18.75%), tislelizumab (11/32, 34.38%), adebrelimab (1/32, 3.12%), and penpulimab (1/32, 3.12%). 59.38% (19/32) had a TPS of 1%–49%. Patients’ chemotherapy regimens that were utilized included albumin-bound paclitaxel + cisplatin (5/32, 15.63%), docetaxel + carboplatin (3/32, 9.38%), pemetrexed disodium + carboplatin (5/32, 15.63%), albumin-bound paclitaxel + carboplatin (14/32, 43.72%), paclitaxel + carboplatin (3/32, 9.38%), albumin-bound paclitaxel + nida platinum (1/32, 3.13%), and pemetrexed disodium + cisplatin (1/32, 3.13%). A total of 19 (59.38%) patients demonstrated a positive response to NCIT treatment. The clinicopathologic details stratified by treatment response status are shown in Table 1. A detailed statistical analysis was conducted, and no statistically significant differences were observed between the response and non-response groups for gender, age, smoking index, KPS, pathological stage, immunotherapy regimen, and TPS (p ≥ 0.05). However, a statistically significant difference was identified between the two groups about histology type (p = 0.029) and chemotherapy regimens (p = 0.041).

**TABLE 1:** Clinicopathological characteristics of the patients with non-small cell lung cancer received combined neoadjuvant chemotherapy and immunotherapy at baseline.

Characteristics	Total, n = 32 (%)	No MPR, non-response, n = 13 (%)	MPR, response, n = 19 (%)	p*-*value^a^
Gender				0.374
Male	27 (84.38)	10 (37.04)	17 (62.96)	
Female	5 (15.62)	3 (60.00)	2 (40.00)	
Age				0.491
≤65	15 (46.88)	5 (33.33)	10 (66.67)	
>65	17 (53.12)	8 (47.06)	9 (52.94)	
Smoking index^b^				0.713
≤400	12 (37.50)	4 (33.33)	8 (66.67)	
>400	20 (62.50)	9 (45.00)	11 (55.00)	
KPS^c^				0.374
100	2 (6.24)	0 (0.00)	2 (100.00)	
90	19 (59.38)	7 (36.84)	12 (63.16)	
80	11 (34.38)	6 (54.55)	5 (45.45)	
Histology type				**0.029**
LUAD	6 (18.75)	5 (83.33)	1 (16.67)	
LUSC	26 (81.25)	8 (30.77)	18 (69.23)	
Pathologic stage (AJCC 8th)				1.000
IIA	1 (3.12)	0 (0.00)	1 (100.00)	
IIB	8 (25.00)	3 (37.50)	5 (62.50)	
IIIA	13 (40.63)	6 (46.15)	7 (53.85)	
IIIB	10 (31.25)	4 (40.00)	6 (60.00)	
Immunotherapy regimens				0.411
Sintilimab	7 (21.88)	2 (28.57)	5 (71.43)	
Toripalimab	6 (18.75)	3 (50.00)	3 (50.00)	
Camrelizumab	6 (18.75)	4 (66.67)	2 (33.33)	
Tislelizumab	11 (34.38)	3 (27.27)	8 (72.73)	
Adebrelimab	1 (3.12)	0 (0.00)	1 (100.00)	
Penpulimab	1 (3.12)	1 (100.00)	0 (0.00)	
TPS^d^				0.899
<1%	1 (3.13)	0 (0.00)	1 (100.00)	
1%–49%	19 (59.38)	9 (47.37)	10 (52.63)	
≥50%	11 (34.38)	4 (36.36)	7 (63.64)	
Unknown	1 (3.13)	0 (0.00)	1 (100.00)	
Chemotherapy regimens				**0.041**
albumin-bound paclitaxel + cisplatin	5 (15.63)	0 (0.00)	5 (100.00)	
docetaxel + carboplatin	3 (9.38)	2 (66.67)	1 (33.33)	
pemetrexed disodium+ carboplatin	5 (15.63)	4 (80.00)	1 (20.00)	
albumin-bound paclitaxel + carboplatin	14 (43.72)	4 (28.57)	10 (71.43)	
paclitaxel + carboplatin	3 (9.38)	2 (66.67)	1 (33.33)	
albumin-bound paclitaxel + nida platinum	1 (3.13)	0 (0.00)	1 (100.00)	
pemetrexed disodium + cisplatin	1 (3.13)	1 (100.00)	0 (0.00)	

Abbreviations: MPR, major pathological response; LUSC, lung squamous cell carcinoma; LUAD, lung adenocarcinoma; KPS, Karnofsky performance status; PD-1, programmed cell death protein-1; AJCC, The American Joint Committee on Cancer.

^a^ p-values for gender, age, smoking index, KPS, histology type, pathologic stage, Immunotherapy regimens, TPS, and Chemotherapy regimens were calculated using Fisher’s exact test.

^b^ Smoking index = number of cigarettes smoked per day × year(s).

^c^ KPS were scored using the Karnofsky (Kahn, KPS, percentile) functional status scale.

^d^ TPS defined as the number of tumor cells positive for PD-L1 membrane staining of any intensity/total number of tumor cells x 100%.

Bold values indicate the significantly different clinicopathological characteristics between patients with response and non-response.

### Tumor immune microenvironment reshaped by neoadjuvant chemoimmunotherapy

The cellular composition of the tumor microenvironment changed significantly after neoadjuvant chemoimmunotherapy in patients with non-small cell lung cancer.

The proportion of tumor cells among all cell types exhibited significant reductions in the overall (41% vs. 19%, p < 0.001) and response groups (42% vs. 10%, p < 0.001), with no change observed in the non-response group. Similarly, CD8^+^ T cell proportions declined in overall and response groups but remained stable in the non-response group. Analysis of CD8^+^ T cell subpopulations revealed divergent trends: CD8^+^ T_rm_ proportions increased within the CD8^+^ T cell population in overall (36% vs. 52%, p = 0.017) and response groups (36% vs. 54%, p = 0.033), but showed no alterations in non-response group; among its subsets, T_rm-pre_ rose in both overall (11% vs. 25%, p = 0.001) and response groups (10% vs. 27%, p = 0.004), and T_rm-dys_ demonstrated significant declines in all groups (overall, response, and non-response) (40% vs. 15%, p < 0.001; 37% vs. 15%, p = 0.001; 44% vs. 14%, p = 0.037), but T_rm-cyt_ showed no alterations in all groups. CD8^+^ T_bys_ proportions decreased in the overall (64% vs. 48%, p = 0.017) and response groups (64% vs. 46%, p = 0.033), yet its subset T_bys-pre_ (14% vs. 27%, p = 0.011) displayed a marked increase in the overall group; T_bys-dys_ exhibited a pronounced decrease in the overall (10% vs. 4%, p = 0.008) and response groups (9% vs. 3%, p = 0.011). For CD4^+^ T cells, their overall proportion increase significantly in overall (27% vs. 37%, p = 0.021) and response groups (27% vs. 45%, p = 0.002) but remained unchanged in non-response group, with CD4^+^ T_con_ showing robust expansion in all groups (88% vs. 95%, p < 0.001; 85% vs. 95%, p < 0.001; 92% vs. 96%, p = 0.016) and CD4^+^ T_reg_ displaying sustained reductions universally (12% vs. 5%, p < 0.001; 15% vs. 5%, p < 0.001; 8% vs. 4%, p = 0.016) (Supplementary Figure S3).

### Intratumoral CD8^+^ T_rm-dys_ and CD8^+^ T_bys-cyt_ decreased and CD4^+^ T_con_ increased in patients with major pathologic response after treatment

**FIGURE 2:**
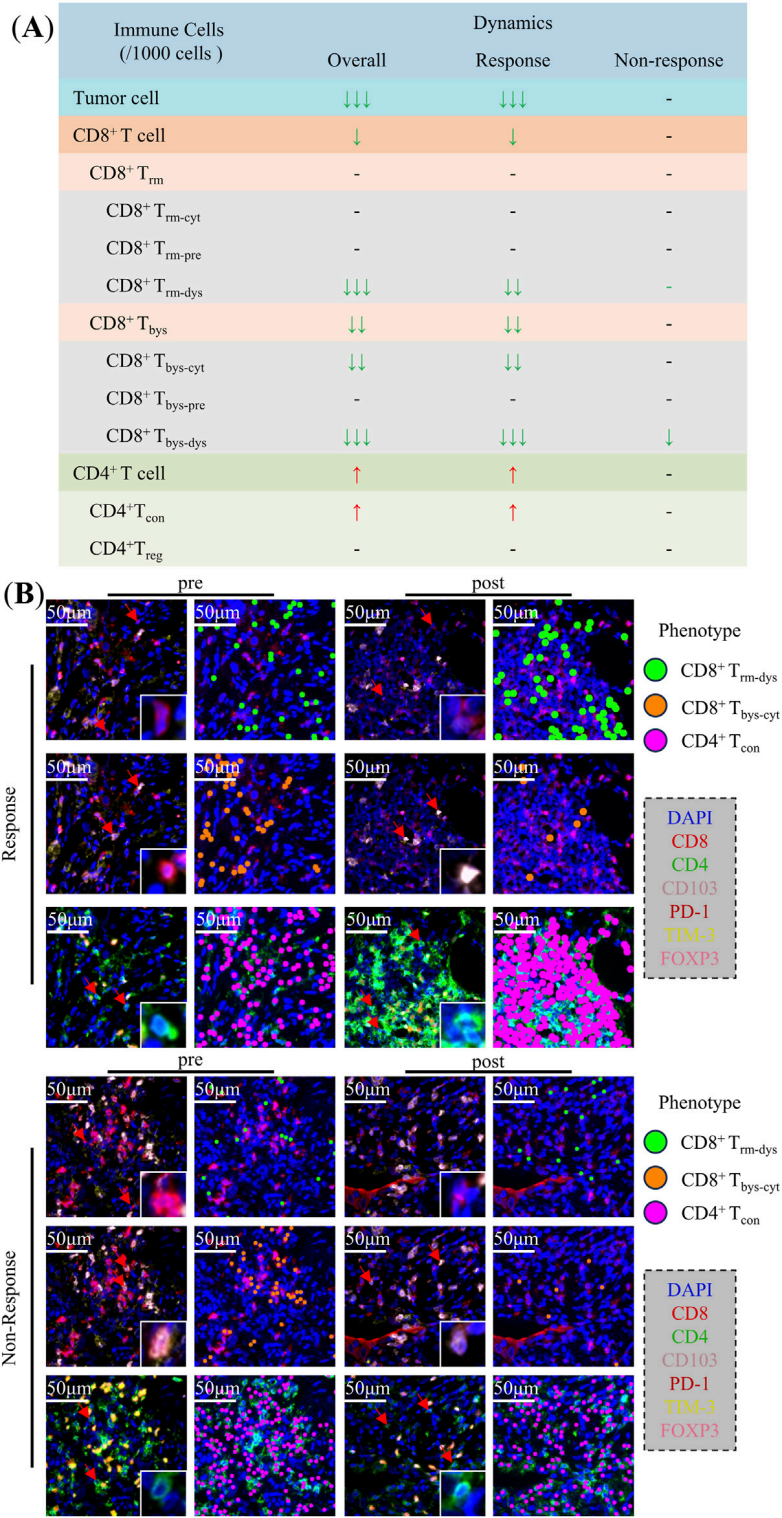
Changes in the Tumor immune microenvironment of non-small cell lung cancer during neoadjuvant chemoimmunotherapy. **(A)** Changes of tumor cells, CD8^+^ T cells, CD4^+^ T cells, and their subpopulations in the overall, response, and non-response groups. **(B)** Typical multiplex immunofluorescence images and dot-style visualizations of CD8^+^ T_rm-dys_, CD8^+^ T_bys-cyt_, and CD4^+^ T_con_ in the response and non-response groups pre and post neoadjuvant chemoimmunotherapy.

In this study, we analyzed images of the immune cells in patients with NSCLC pre- and post-NCIT. We then identified and quantified the density of CD8^+^ T and CD4^+^ T cells and their subpopulations within TIME based on the co-expression of different biomarkers. Finally, we analyzed their density changes during treatment in the overall, response and non-response groups (Figure 2A).

**FIGURE 3:**
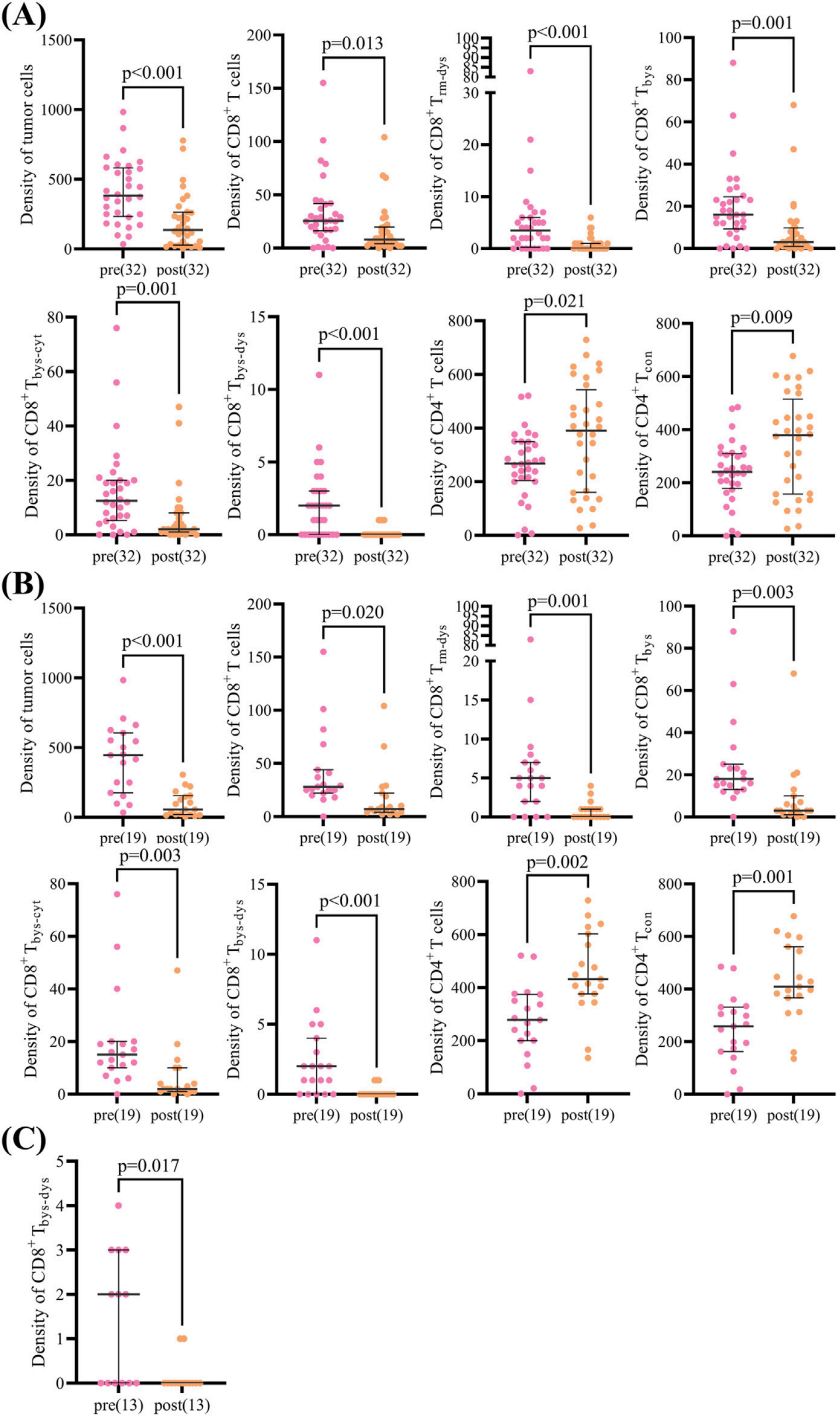
Scatter plot of paired cell density pre and post neoadjuvant chemoimmunotherapy in patients with non-small cell lung cancer. **(A)** Scatter plot showing differences in paired cell density pre and post neoadjuvant chemoimmunotherapy in the overall group (32 patients). **(B)** Scatter plot showing differences in paired cell density pre and post neoadjuvant chemoimmunotherapy in the response group (19 patients). **(C) **Scatter plot showing differences in paired cell density pre and post neoadjuvant chemoimmunotherapy in the non-response group (13 patients). The Wilcoxon signed-rank test was used to analyze differences in paired cell density between pre- and post-neoadjuvant chemoimmunotherapy.

The analysis of TIME in NSCLC patients overall demonstrated that CD8^+^ T cells [26 (16.42) vs. 8 (4.20), p = 0.013], CD8^+^ T_rm-dys_ [4 (0.6) vs. 0 (0.1), p < 0.001], CD8^+^ T_bys_ [16 (9.25) vs. 3 (1.10), p = 0.001], CD8^+^ T_bys-cyt_ [13 (5.20) vs. 2 (1.8), p = 0.001], and CD8^+^ T_bys-dys_ [2 (0.3) vs. 0 (0.0), p < 0.001] were reduced in density after NCIT. Conversely, the densities of CD4^+^ T cells [269 (205,350) vs. 391 (161,543), p = 0.021], and CD4^+^ T_con_ [241 (179,310) vs. 379 (158,515), p = 0.009] were significantly higher (Supplementary Table 1). The response group exhibited a comparable trend of changes in NCIT as the overall NSCLC patients, with significant increases in the density of CD8^+^ T cells [28 (22.44) vs. 7 (4.22), p = 0.020], CD8^+^ T_rm-dys_ [5 (2.7) vs. 0 (0.1), p = 0.001], CD8^+^ T_bys_ [18 (13.25) vs. 3 (1.10), p = 0.003], CD8^+^ T_bys-cyt_ [15 (10.20) vs. 2 (1.10), p = 0.003], and CD8^+^ T_bys-dys_ [2 (0.4) vs. 0 (0.0), p < 0.001] had reduced densities, whereas CD4^+^ T cells [278 (200,374) vs. 432 (376,603), p = 0.002], CD4^+^ T_con_ [258 (162,331) vs. 409 (366,561), p = 0.001] had significantly higher densities (Supplementary Table 2). Conversely, within the TIME of the non-response group of NSCLC patients, solely the alteration in CD8^+^ T_bys-dys_ [2 (0.3) vs. 0 (0.0), p = 0.017] density was statistically significant after NCIT (Figure 3; Supplementary Table 3). Typical mIF images of CD8^+^ T_rm-dys_, CD8^+^ T_bys-cyt_, and CD4^+^ T_con_ before and after NCIT in the response and non-response groups are shown in Figure 2B.

The preceding analysis demonstrated a decline in CD8^+^ T_rm-dys_ and CD8^+^ T_bys-cyt_, accompanied by an increase in CD4^+^ T_con_, within the TIME of NSCLC patients following NCIT. These alterations were predominantly observed in the response group.

### Increase in CD4^+^ T_con_ during treatment is favorable for a better response to neoadjuvant chemoimmunotherapy for NSCLC

**FIGURE 4:**
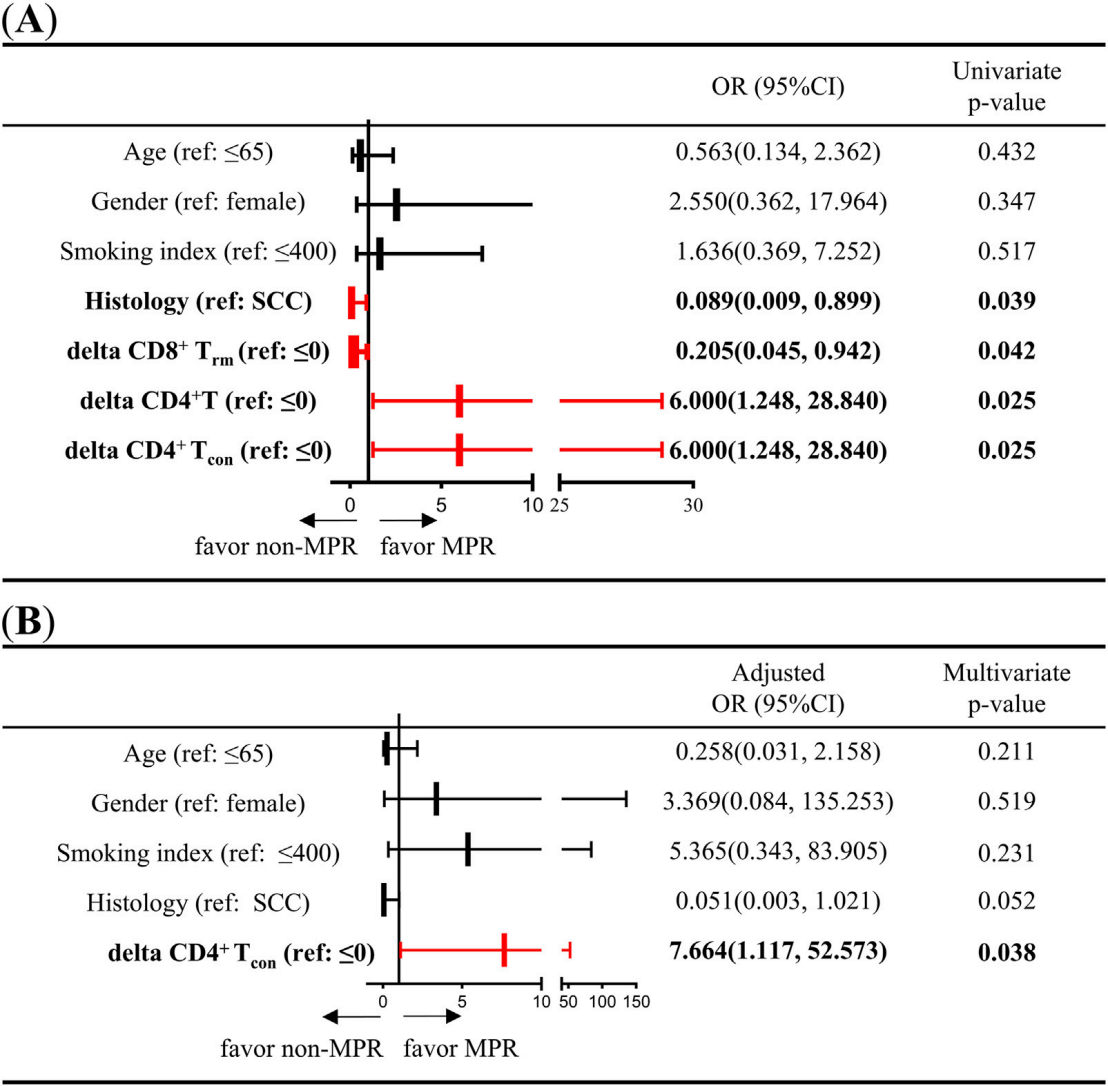
Univariate and multivariate logistic regression analyses of the association between clinicopathological characteristics (age, sex, smoking index, and pathology type) and changes in CD4^+^ T cells and CD4^+^ T_con_ cell densities during neoadjuvant chemoimmunotherapy. **(A)** Univariate logistic regression was used to analyze the association between clinicopathological characteristics (age, sex, smoking index, and histology type), changes in CD8^+^ T_rm_, CD4^+^ T cells, and CD4^+^ T_con_ densities, and response to neoadjuvant chemoimmunotherapy. **(B)** Multivariate logistic regression was used to analyze the association between clinicopathological characteristics (age, sex, smoking index, and histology type), CD4^+^ T_con_ density, and response to neoadjuvant chemoimmunotherapy. Delta = post-treatment minus pre-treatment.

To further analyze the association between changes in immune cells during NCIT and treatment response, the delta parameter (delta = post-treatment minus pre-treatment) was introduced to represent the changes in immune cells during NCIT. Additionally, using ‘0’ as the cutoff value, the delta T cells were divided into high (>0) and low (≤0) groups. Initially, the association between clinicopathological characteristics (age, gender, smoking index, and histological type), immune cell changes during NCIT, and treatment response was assessed using univariate logistic analyses (Supplementary Table 4). These analyses indicate that adenocarcinoma is more favorable to non-MPR than squamous cell carcinoma (p = 0.039, OR = 0.089, 95% CI = 0.009–0.899). Furthermore, the occurrence of MPR is more likely to happen in high delta CD4^+^ T cells and high delta CD4^+^ T_con_ groups (p = 0.025, OR = 6.000, 95% CI = 1.248–28.840; p = 0.025, OR = 6.000, 95% CI = 1.248–28.840), but patients with non-small cell lung cancer (NSCLC) exhibiting low levels of delta CD8^+^ T_rm_ cells are more likely to achieve MPR (Figure 4A). Subsequently, a multivariate logistic regression analysis was performed. This analysis demonstrated that elevated levels of delta CD4^+^ T_con_ (>0) were more likely to be associated with MPR during NCIT (p = 0.038, OR = 0.13, 95%CI = 0.02–0.90) (Figure 4B). The results obtained using delta CD4^+^ T cells were identical to those obtained using delta CD4^+^ T_con_ cells, but histological type and delta CD8^+^ T_rm_ are not associated with treatment response (Supplementary Table 5). The aforementioned results suggest that NSCLC patients with elevated CD4^+^ T_con_ within TIME responded better to treatment during NCIT.

### Trend observed in CD4^+^ T_con_ is similar to HIF-1α during neoadjuvant chemoimmunotherapy

**FIGURE 5:**
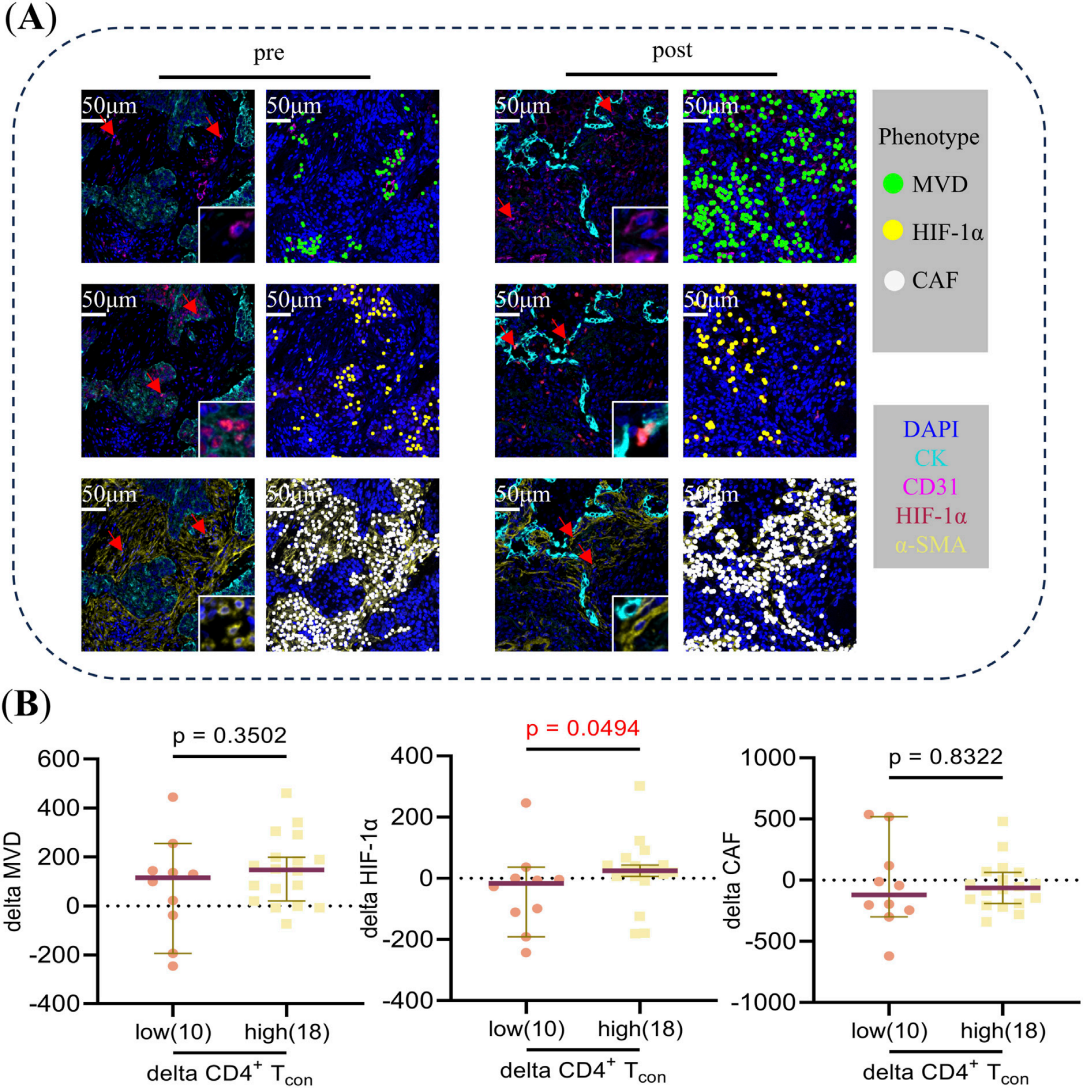
**(A)** Typical multiplex immunofluorescence images and dot-style visualizations of MVD, HIF-1α, and CAF pre and post neoadjuvant chemoimmunotherapy. **(B)** The Mann-Whitney U test was used to analyze differences in delta MVD, HIF-1α, and CAF between CD4^+^ T_con_ cells in the high versus low groups during neoadjuvant chemoimmunotherapy. Delta = post-treatment minus pre-treatment. Using “0” as the cut-off value to distinguish between the high (>0) and low (≤0) groups.

An analysis was conducted to ascertain the disparities in delta MVD, delta HIF-1α, and delta CAF between patients with elevated and diminished delta CD4^+^ T and delta CD4^+^ T_con._ Of the 32 patients enrolled in panel 2, mIF images were available for subsequent analysis in 31 and 29 patients pre- and post-NCIT, respectively. Furthermore, only 28 patients had mIF images available for both the pre- and post-NCIT. Figure 5A shows the representative images of MVD, HIF-1α, and CAF pre- and post-NCIT. Changes in HIF-1α levels within the tumor followed the same trend as changes in CD4^+^ T_con_ (p = 0.0494), but no such trend was observed between MVD changes and CAF changes (Figure 5B). The results obtained using delta CD4^+^ T cells were identical to those obtained using delta CD4^+^ T_con_ cells. Furthermore, no differences in MVD, HIF-1α, and CAF infiltration were observed between patients with high versus low CD4^+^ T and CD4^+^ T_con_ before and after treatment (Supplementary Figure 4). These results suggest that elevated HIF-1α may favor the proliferation of CD4^+^ T_con_.

## Discussion

In this study, we used mIF to discover changes in T cell subset density associated with treatment response within TIME during NCIT. Key findings include: patients in the response group had reduced CD8^+^ T_rm-dys_ and CD8^+^ T_bys-cyt_ and increased CD4^+^ T_con_. In addition, high delta CD4^+^ T_con_ cells are more favorable for MPR, exhibiting a similar trend to changes in HIF-1α.

Previous studies have shown that CD8^+^ T cells were the core of antitumor immunity. However, due to clonal attrition induced by apoptosis, the presence of tumor-infiltrating T cells was insufficient to induce tumor rejection. Unlike effector T cells, long-lived memory T cells persist in chronic tumors and participate in tumor immune surveillance. Recent studies reveal that T cells within the tumor immune environment (TIME) constitute a complex population with multiple heterogeneous subpopulations. Patients who achieve an MPR following immunotherapy exhibit reduced densities of dysfunctional T cells, demonstrating that immunotherapy can enhance the anti-tumor effects by reversing the dysfunctional state of immune cells [24, 41, 42]. Our study confirms a significant reduction in the density of dysfunctional memory CD8^+^ T_rm-dys_ cells post-treatment in MPR patients. While we did not demonstrate that immunotherapy reverses the function of CD8^+^ T_rm-dys_ cells, our findings are consistent with previous studies, both indicating that a reduced density of CD8^+^ T_rm-dys_ cells is associated with favorable treatment responses.

Tumour antigen-specific T cells form the basis of effective anti-cancer immunity and play a central role in cancer immunotherapy [16]. Within the TIME, however, these cells constitute only a tiny fraction of tumor-infiltrating T cells and are prone to functional exhaustion. This prevents the body from effectively eliminating tumor cells. The TIME harbours a large and highly heterogeneous population of T_bys_ cells that recognize various viruses previously encountered by the host, yet fail to recognize tumor antigens. T_bys_ cells can be activated during viral reinfection, exhibiting partial antitumor effects and synergising with immune checkpoint blockade [13–15, 43]. Our study confirms that, post-treatment, the density of cytotoxic bystander T_bys-cyt_ cells is significantly reduced in MPR patients. While we did not determine the role of T_bys-cyt_ cells in predicting treatment response, our findings suggest that these cells are reduced during NCIT therapy due to their reactivation and subsequent involvement in anti-tumor activity.

While effector CD8^+^ T cells activated by antigen-presenting cells have long been considered the primary immune target because of their unique cytotoxicity, several studies in recent years have identified cytotoxic CD4^+^ T cells with a cytotoxicity program that can directly kill cancer cells [27]. Tomasz Ahrends et al, using anti-tumor vaccine mouse models, have shown at the molecular level that CD4^+^ T cells promote the migration and recognition of cytotoxic T cells towards tumor cells, and thus kill tumor cells, by down-regulating the expression of co-inhibitory receptors that affect cytotoxic T cell activity and by up-regulating the expression of multiple chemokine receptors on cytotoxic T cells [28]. Currently, a large number of studies have demonstrated that Treg is an important component of the immunosuppressive microenvironment, suppressing cytotoxic T-cell responses through the production of inhibitory cytokines or by indirectly influencing the status and function of dendritic cells and other (innate) immune cell types [44]. CD4^+^ T_con_ cells are a group of subpopulations such as TH1, TH2, and TH17 cell clusters that can support the activation and value-adding of CD8^+^ T cells by secreting a variety of immunomodulatory cytokines, such as IL-2 [45]. T_con_ has been shown to mediate adaptive immune responses and Treg cells suppress excessive immune responses to protect the body from autoimmune and inflammatory diseases [45, 46], both representing a state of pro-immunity and immunosuppression that is balanced in healthy individuals and immunosuppressed in TIME due to an imbalance between the two. Immune checkpoint blockade exerts anti-tumor effects by reversing the suppressed state of the TIME. Our study found that following NCIT treatment, CD4^+^ T_con_ cell density significantly increased in MPR patients. Although we did not demonstrate the specific antitumor effects of CD4^+^ T_con_ cells, our findings indicate that increased CD4^+^ T_con_ during NCIT is associated with a more favorable treatment response.

HIF-1α has long been recognized as a key regulator of cellular adaptive responses to hypoxia. HIF-1α has been shown to control T cell effector function and anti-tumor immune response in hypoxic T cells through its regulatory role on IFN-γ production, whereas HIF-1α deficiency leads to T cell resistance to immune checkpoint blockers [47]. Numerous studies have demonstrated that HIF-1α can induce T_h_17 cell differentiation, promote FOXP3 degradation, and compromise the stability of Treg cells [48, 49]. Furthermore, animal experiments have provided additional evidence that mice with HIF-1α-deficient T cells are incapable of mounting a robust Th17 response while exhibiting an increased population of Treg cells [50, 51]. These findings suggest that elevated HIF-1α expression leads to a reduction in Treg cells and an increase in T_con_ cells, which is consistent with our experimental results. Based on our findings, we propose that HIF-1α may potentiate anti-tumor immune responses through its regulatory role in promoting CD4^+^ T_con_ proliferation and activation.

This study has the following limitations. First, the limited availability of paired samples resulted in a small number of enrolled patients, necessitating multiple comparisons without any correction for many cell subsets. We plan to expand the sample size in future follow-up studies to further validate the findings. Second, this study focused solely on CD4^+^ T_con_ cells without analyzing their subpopulations. It is also necessary to evaluate the distinct functional states of CD4^+^ T_con_ cells. Finally, although there is literature supporting the use of CD103^-^ to mark bystander CD8^+^ T cells for histological applications, future studies should incorporate TCR sequencing to determine antigen specificity and better characterise such cell populations. Additionally, we observed an association between HIF-1α expression and CD4^+^ T_con_ infiltration, though the underlying mechanisms warrant further investigation.

In conclusion, our study used paired pre- and post-treatment specimens to analyze T-cell subpopulations of different functional states that changed during treatment. Increased CD4^+^ T_con_ is favorable for treatment response. Targeting HIF-1α may provide a new therapeutic strategy for NSCLC.

## Data Availability

The raw data supporting the conclusions of this article will be made available by the authors, without undue reservation.

## Supplementary material

SUPPLEMENTARY FIGURE 1 Flowchart of the patient population in this study.

SUPPLEMENTARY FIGURE 2 Construction, staining, and spot selection of tissue microarrays (TMA).

SUPPLEMENTARY FIGURE 3
**(A)** Changes in the proportion of cellular components within the tumor microenvironment pre- and post-neoadjuvant chemoimmunotherapy in the overall group. **(B)** Changes in the proportion of cellular components within the tumor microenvironment pre- and post-neoadjuvant chemoimmunotherapy in the response group. **(C)** Changes in the proportion of cellular components within the tumor microenvironment pre- and post-neoadjuvant chemoimmunotherapy in the non-response group. The figure shows the average proportion of cells across all patients. The Wilcoxon Signed-Rank test was used to analyze the changes in the proportion of cellular components in the overall group, response group, and non-response group. The arrow symbols at the back of the legend indicate statistically significant differences before and after treatment (p < 0.05).

SUPPLEMENTARY FIGURE 4
**(A)** The Mann-Whitney U test was used to analyse the differences in delta MVD, HIF-1α, and CAF between CD4^+^ T cells in the high versus low groups before neoadjuvant chemoimmunotherapy; **(B)** The Mann-Whitney U test was used to analyse the differences in delta MVD, HIF-1α, and CAF between CD4^+^ T cells in the high versus low groups after neoadjuvant chemoimmunotherapy; **(C)** The Mann-Whitney U test was used to analyse the differences in delta MVD, HIF-1α, and CAF between CD4^+^ T_con_ cell in the high versus low groups before neoadjuvant chemoimmunotherapy; **(D)** The Mann-Whitney U test was used to analyse the differences in delta MVD, HIF-1α, and CAF between CD4^+^ T_con_ cell in the high versus low groups after neoadjuvant chemoimmunotherapy.

## Abbreviations

NSCLC, Non-small cell lung cancer; mIF, Multiplex immunofluorescence; MPR, Major pathologic response; pCR, Pathologic complete response; TIME, Tumor immune microenvironment; CD8^+^ T_rm_, CD8^+^ resident memory T cells; CD8^+^ T_rm-cyt_ - Cytotoxic CD8^+^ resident memory T cells; CD8^+^ T_rm-pre_, Pre-dysfunctional CD8^+^ resident memory T cells; CD8^+^ T_rm-dys_, Dysfunctional CD8^+^ resident memory T cells; CD8^+^ T_bys_, CD8^+^ bystander T cells; CD8^+^ T_bys-cyt_, Cytotoxic CD8^+^ bystander T cells; CD8^+^ T_bys-pre_, Pre-dysfunctional CD8^+^ bystander T cells; CD8^+^ T_bys-dys_, Dysfunctional CD8^+^ bystander T cells; CD4^+^ T_con_, Conventional CD4^+^ T cells; CD4^+^ T_reg_, Regulatory CD4^+^ T cells; NCIT, Neoadjuvant chemoimmunotherapy; FFPE, Formalin-fixed paraffin-embedded; MVD, Microvessel density; HIF-1α, Hypoxia inducible factor-1α; CAFs, Cancer-associated fibroblasts; TILs, Tumor infiltrating lymphocytes; ROIs, Regions of interests.
