# Supplementary material for: Synergistic changes in bystander CD8 and conventional CD4 T cells during neoadjuvant chemoimmunotherapy for non-small cell lung cancer reveal treatment response
Source: Pathol Oncol Res. 2025 Oct 28;31:1612229. doi: 10.3389/pore.2025.1612229 (PMC12602375; doi:10.3389/pore.2025.1612229)
Supplement: Supplementary file 2 [file Table1.docx]

| Cell subsets  (cell/1000) | pre-Treatment median (Q1, Q3) | post-Treatment median (Q1, Q3) | p*-*value |
| --- | --- | --- | --- |
| **Tumor cell** | **383(233,582)** | **137(28,264)** | **<0.001** |
| **CD8^+^ T cells** | **26(16,42)** | **8(4,20)** | **0.013** |
| CD8^+^ T_rm_ | 10(5,15) | 3(2,9) | 0.106 |
| CD8^+^ T_rm-cyt_ | 6(1,10) | 2(1,6) | 0.156 |
| CD8^+^ T_rm-pre_ | 1(0,2) | 1(0,2) | 0.678 |
| **CD8^+^ T_rm-dys_** | **4(0,6)** | **0(0,1)** | **<0.001** |
| **CD8^+^ T_bys_** | **16(9,25)** | **3(1,10)** | **0.001** |
| **CD8^+^ T_bys-cyt_** | **13(5,20)** | **2(1,8)** | **0.001** |
| CD8^+^ T_bys-pre_ | 1(0,3) | 1(0,3) | 0.796 |
| **CD8^+^ T_bys-dys_** | **2(0,3)** | **0(0,0)** | **<0.001** |
| **CD4^+^ T cells** | **269(205,350)** | **391(161,543)** | **0.021** |
| **CD4^+^ T_con_** | **241(179,310)** | **379(158,515)** | **0.009** |
| CD4^+^ T_reg_ | 27(11,41) | 18(4,31) | 0.088 |

**Supplementary Table 1. Changes in the tumor immune microenvironment of overall group after neoadjuvant chemoimmunotherapy**

The data presentation shows the median and interquartile range of cell density for each cell subset per 1000 cells. Boldface type indicates statistical significance on paired nonparametric Wilcoxon test.
