# Supplementary material for: Synergistic changes in bystander CD8 and conventional CD4 T cells during neoadjuvant chemoimmunotherapy for non-small cell lung cancer reveal treatment response
Source: Pathol Oncol Res. 2025 Oct 28;31:1612229. doi: 10.3389/pore.2025.1612229 (PMC12602375; doi:10.3389/pore.2025.1612229)
Supplement: Supplementary file 6 [file Table2.docx]

| Cell subsets  (cell/1000) | pre-Treatment median (Q1, Q3) | post-Treatment median (Q1, Q3) | p*-*value |
| --- | --- | --- | --- |
| **Tumor cell** | **445(175,606)** | **56(21,155)** | **<0.001** |
| **CD8^+^ T cells** | **28(22,44)** | **7(4,22)** | **0.020** |
| CD8^+^ T_rm_ | 13(7,19) | 3(2,7) | 0.064 |
| CD8^+^ T_rm-cyt_ | 6(3,10) | 2(1,5) | 0.073 |
| CD8^+^ T_rm-pre_ | 1(0,2) | 1(0,2) | 0.968 |
| **CD8^+^ T_rm-dys_** | **5(2,7)** | **0(0,1)** | **0.001** |
| **CD8^+^ T_bys_** | **18(13,25)** | **3(1,10)** | **0.003** |
| **CD8^+^ T_bys-cyt_** | **15(10,20)** | **2(1,10)** | **0.003** |
| CD8^+^ T_bys-pre_ | 2(0,3) | 1(0,3) | 0.440 |
| **CD8^+^ T_bys-dys_** | **2(0,4)** | **0(0,0)** | **<0.001** |
| **CD4^+^ T cells** | **278(200,374)** | **432(376,603)** | **0.002** |
| **CD4^+^ T_con_** | **258(162,331)** | **409(366,561)** | **0.001** |
| CD4^+^ T_reg_ | 32(17,42) | 24(11,42) | 0.481 |

**Supplementary Table 2. Changes in the tumor immune microenvironment of response group after** **neoadjuvant chemoimmunotherapy**

The data presentation shows the median and interquartile range of cell density for each cell subset per 1000 cells. Boldface type indicates statistical significance on paired nonparametric Wilcoxon test.
