# Supplementary material for: Synergistic changes in bystander CD8 and conventional CD4 T cells during neoadjuvant chemoimmunotherapy for non-small cell lung cancer reveal treatment response
Source: Pathol Oncol Res. 2025 Oct 28;31:1612229. doi: 10.3389/pore.2025.1612229 (PMC12602375; doi:10.3389/pore.2025.1612229)
Supplement: Supplementary file 7 [file Table5.docx]

**Supplementary Table 5. Multivariate logistic analyses of the association between clinicopathological characteristics, delta CD8^+^ T_rm_ and delta CD4^+^ T cells, and response to** **neoadjuvant chemoimmunotherapy**

| clinicopathological characteristics | Multivariate  p*-*value | OR | 95%CI |
| --- | --- | --- | --- |
| Age |  |  |  |
| ≤65 |  | 1(ref) |  |
| >65 | 0.057 | 0.071 | 0.005,1.079 |
| Gender |  |  |  |
| female |  | 1(ref) |  |
| Male | 0.9998 | 2603981331.834 |  |
| Smoking index |  |  |  |
| ≤400 |  | 1(ref) |  |
| >400 | 0.967 | 0.924 | 0.023,37.541 |
| Histology |  |  |  |
| LUSC |  | 1(ref) |  |
| LUAD | 0.997 | 0.000 |  |
| delta CD8^+^ T_rm_ |  |  |  |
| ≤0 |  | 1(ref) |  |
| >0 | 0.997 | 0.000 |  |
| Age |  |  |  |
| ≤65 |  | 1(ref) |  |
| >65 | 0.211 | 0.258 | 0.031,2.158 |
| Gender |  |  |  |
| female |  | 1(ref) |  |
| Male | 0.519 | 3.369 | 0.084,135.253 |
| Smoking index |  |  |  |
| ≤400 |  | 1(ref) |  |
| >400 | 0.231 | 5.365 | 0.343,83.905 |
| Histology |  |  |  |
| SCC |  | 1(ref) |  |
| ADC | 0.052 | 0.051 | 0.003,1.021 |
| **delta CD4 T cells** |  |  |  |
| **≤0** |  | 1(ref) |  |
| **>0** | **0.038** | **7.664** | **1.117,552.573** |

The data presentation shows the median and interquartile range of cell density for each cell subset per 1000 cells. Delta = post-treatment minus pre-treatment. Boldface type indicates statistical significance on Univariate logistic analyses. OR = odds ratio; ref = reference.
