# Supplementary material for: Synergistic changes in bystander CD8 and conventional CD4 T cells during neoadjuvant chemoimmunotherapy for non-small cell lung cancer reveal treatment response
Source: Pathol Oncol Res. 2025 Oct 28;31:1612229. doi: 10.3389/pore.2025.1612229 (PMC12602375; doi:10.3389/pore.2025.1612229)
Supplement: Supplementary file 8 [file Table3.docx]

| Cell subsets  (cell/1000) | pre-Treatment median (Q1, Q3) | post-Treatment median (Q1, Q3) | p*-*value |
| --- | --- | --- | --- |
| Tumor cell | 359(245,580) | 266(170,473) | 0.311 |
| CD8^+^ T cells | 17(1,37) | 11(2,18) | 0.345 |
| CD8^+^ T_rm_ | 5(1,13) | 3(1,13) | 0.959 |
| CD8^+^ T_rm-cyt_ | 3(0,6) | 2(0,9) | 0.759 |
| CD8^+^ T_rm-pre_ | 1(0,2) | 1(0,2) | 0.559 |
| CD8^+^ T_rm-dys_ | 2(0,4) | 0(0,2) | 0.084 |
| CD8^+^ T_bys_ | 10(1,26) | 4(1,11) | 0.223 |
| CD8^+^ T_bys-cyt_ | 7(1,22) | 3(1,8) | 0.245 |
| CD8^+^ T_bys-pre_ | 1(0,2) | 1(0,3) | 0.473 |
| **CD8^+^ T_bys-dys_** | **2(0,3)** | **0(0,0)** | **0.017** |
| CD4^+^ T cells | 262(216,329) | 159(97,375) | 0.807 |
| CD4^+^ T_con_ | 234(207,281) | 157(95,357) | 0.889 |
| CD4^+^ T_reg_ | 14(9,41) | 5(2,18) | 0.060 |

**Supplementary Table 3. Changes in the tumor immune microenvironment of non-response group after** **neoadjuvant chemoimmunotherapy**

The data presentation shows the median and interquartile range of cell density for each cell subset per 1000 cells. Boldface type indicates statistical significance on paired nonparametric Wilcoxon test.
