# Supplementary material for: Synergistic changes in bystander CD8 and conventional CD4 T cells during neoadjuvant chemoimmunotherapy for non-small cell lung cancer reveal treatment response
Source: Pathol Oncol Res. 2025 Oct 28;31:1612229. doi: 10.3389/pore.2025.1612229 (PMC12602375; doi:10.3389/pore.2025.1612229)
Supplement: Supplementary file 9 [file Table4.docx]

**Supplementary Table 4. Univariate logistic analyses of the association between clinicopathological characteristics, the functional subpopulations of CD8^+^ T and CD4^+^ T cells, and response to** **neoadjuvant chemoimmunotherapy**

| clinicopathological characteristics | Univariate  p*-*value | OR | 95%CI |
| --- | --- | --- | --- |
| Age |  |  |  |
| ≤65 |  | 1(ref) |  |
| >65 | 0.432 | 0.563 | 0.134,2.362 |
| Gender |  |  |  |
| female |  | 1(ref) |  |
| Male | 0.347 | 2.550 | 0.362,17.964 |
| Smoking index |  |  |  |
| ≤400 |  | 1(ref) |  |
| >400 | 0.517 | 1.636 | 0.369,7.252 |
| **Histology** |  |  |  |
| **LUSC** |  | 1(ref) |  |
| **LUAD** | **0.039** | **0.089** | **0.009,0.889** |
| delta tumor cells |  |  |  |
| ≤0 |  | 1(ref) |  |
| >0 | 0.156 | 0.300 | 0.057,1.584 |
| delta CD8^+^ T cells |  |  |  |
| ≤0 |  | 1(ref) |  |
| >0 | 0.783 | 0.804 | 0.169,3.819 |
| **delta CD8^+^ T_rm_** |  |  |  |
| **≤0** |  | 1(ref) |  |
| **>0** | **0.042** | **0.205** | **0.045,0.942** |
| delta CD8^+^ T_rm-cyt_ |  |  |  |
| ≤0 |  | 1(ref) |  |
| >0 | 0.099 | 0.288 | 0.066,1.265 |
| delta CD8^+^ T_rm-pre_ |  |  |  |
| ≤0 |  | 1(ref) |  |
| >0 | 0.601 | 0.65 | 0.13,3.26 |
| delta CD8^+^ T_rm-dys_ |  |  |  |
| ≤0 |  | 1(ref) |  |
| >0 | 0.688 | 0.738 | 0.168,3.237 |
| delta CD8^+^ T_bys_ |  |  |  |
| ≤0 |  | 1(ref) |  |
| >0 | 0.156 | 0.3 | 0.057,1.584 |
| delta CD8^+^ T_bys-cyt_ |  |  |  |
| ≤0 |  | 1(ref) |  |
| >0 | 0.07 | 0.219 | 0.042,1.135 |
| delta CD8^+^ T_bys-pre_ |  |  |  |
| ≤0 |  | 1(ref) |  |
| >0 | 0.351 | 0.494 | 0.112,2.175 |
| delta CD8^+^ T_bys-dys_ |  |  |  |
| ≤0 |  | 1(ref) |  |
| >0 | 0.251 | 0.417 | 0.094,1.856 |
| **delta CD4 T cells** |  |  |  |
| **≤0** |  | 1(ref) |  |
| **>0** | **0.025** | **6.000** | **1.248,28.840** |
| **delta CD4^+^ T_con_** |  |  |  |
| **≤0** |  | 1(ref) |  |
| **>0** | **0.025** | **6.000** | **1.248,28.840** |
| delta CD4^+^ T_reg_ |  |  |  |
| ≤0 |  | 1(ref) |  |
| >0 | 0.517 | 1.636 | 0.369,7.252 |

The data presentation shows the median and interquartile range of cell density for each cell subset per 1000 cells. Delta = post-treatment minus pre-treatment. Boldface type indicates statistical significance on Univariate logistic analyses. OR = odds ratio; ref = reference.
